# Effect of Shenfu Injection on Differentiation of Bone Marrow Mesenchymal Stem Cells into Pacemaker-Like Cells and Improvement of Pacing Function of Sinoatrial Node

**DOI:** 10.1155/2022/4299892

**Published:** 2022-02-10

**Authors:** Qi Chen, Liang Kang, Yihua Li, Zina Lin, Qingmin Chu, Yinhe Cai, Wei Wu, Song Wang, Lijin Qing, Xinjun Zhao, Rong Li

**Affiliations:** ^1^Department of Internal Medicine-Cardiovascular, The First Affiliated Hospital of Guangzhou University of Chinese Medicine, Guangzhou, China; ^2^Department of Internal Medicine-Cardiovascular, Shenzhen Hospital of Guangzhou University of Chinese Medicine, Shenzhen, China; ^3^The First Clinical Medical School, Guangzhou University of Chinese Medicine, Guangzhou, China

## Abstract

Sick sinus syndrome (SSS), a complex type of cardiac arrhythmia, is a major health threat to humans. Shenfu injection (SFI), a formula of traditional Chinese medicine (TCM), is effective in improving bradyarrhythmia. However, the underlying mechanism of SFI's therapeutic effect is subject to few systematic investigations. The purpose of the present research is to examine whether SFI can boost the differentiation effectiveness of bone marrow mesenchymal stem cells (BMSCs) into pacemaker-like cells and whether the transplantation of these cells can improve the pacing function of the sinoatrial node (SAN) in a rabbit model of SSS. BMSCs from New Zealand rabbits were extracted, followed by incubation in vitro. The flow cytometry was utilized to identify the expression of CD29, CD44, CD90, and CD105 surface markers. The isolated BMSCs were treated with SFI, and the whole-cell patch-clamp method was performed to detect hyperpolarization-the activated cyclic nucleotide-gated potassium channel 4 (HCN4) channel current activation curve. The SSS rabbit model was established using the formaldehyde wet dressing method, and BMSCs treated with SFI were transplanted into the SAN of the SSS rabbit model. We detected changes in the body-surface electrocardiogram and recorded dynamic heart rate measurements. Furthermore, transplanted SFI-treated BMSCs were subjected to HE staining, TUNEL staining, qPCR, western blotting, immunofluorescence, immunohistochemistry, and enzyme-linked immunosorbent assay to study their characteristics. Our results indicate that the transplantation of SFI-treated BMSCs into the SAN of SSS rabbits improved the pacing function of the SAN. In vitro data showed that SFI induced the proliferation of BMSCs, promoted their differentiation capacity into pacemaker-like cells, and increased the *HCN4* expression in BMSCs. In vivo, the transplantation of SFI treated-BMSCs preserved the function of SAN in SSS rabbits, improved the expression of the *HCN4* gene and gap junction proteins (Cx43 and Cx45), and significantly upregulated the expression of cAMP in the SAN, compared to the SSS model group. In summary, the present research demonstrated that SFI might enhance the differentiation capacity of BMSCs into pacemaker-like cells, hence offering a novel approach for the development of biological pacemakers. Additionally, we confirmed the effectiveness and safety of pacemaker-like cells differentiated from BMSCs in improving the pacing function of the SAN.

## 1. Introduction

Sick sinus syndrome (SSS) comprises various cardiac conditions, such as the abnormal pacing of the sinoatrial node (SAN) or alterations of conduction function, which leads to abnormalities in the heart rhythm (arrhythmias), such as sinus arrest, SAN exit block, and sinus bradycardia. Currently, the most effective treatment of SSS is artificial cardiac pacemaker implantation [1]. However, the implantation of an electronic pacemaker in patients is accompanied by several complications, such as infection, electrode dysfunction, battery exhaustion, and bleeding [2]. Recently, for the purpose of circumventing the limitations and negative impacts of electronic pacemakers, stem cell-based biological pacemakers have been widely investigated so as to initiate the spontaneous activity of formerly quiescent cardiomyocytes [3–5]. Recent investigations have mainly focused on the induction of pacemaker-like cells through genetic reprogramming of stem cells [6]. The hyperpolarization-activated nucleotide-gated channel (HCN4) is the predominant isoform in the murine sinus atrial node (SAN) at both RNA and protein levels [7]. Previous research has reported that bone marrow mesenchymal cells (BMSCs) were transfected with the *HCN4* gene and injected into the subepicardium of dogs to induce a stable biological pacemaker activity in vivo for up to six weeks [8]. As a marker for pacemaker cells of the heart, HCN4 drives the generation of the pacemaker current (*I*_*f*_, also referred to as funny current) and the maintenance of sinus rhythm [9, 10]. Therefore, HCN4 facilitates spontaneous discharge and maintains a stable heart rhythm during the transition between activated and basal cardiometabolic status. A study showed the successful transplantation of a genetic pacemaker via the adenoviral transfer of HCN4 in pigs with complete atrioventricular (AV) block [11]. However, knockout of HCN4 in adult mice or human cause cardiac arrhythmia with a reduction in *I*_*f*_ in the SAN [12, 13].

Shenfu injection (SFI), a known traditional Chinese herbs extract, includes ginsenoside and aconitine. Studies have proved that ginsenoside significantly protects against heart failure and improves cardiac function [14–16]. Aconitine is extracted from Fuzi, and modern pharmacological studies have proved that Fuzi exerts a protective effect on myocardial cells and could activate *α* and *β*-adrenergic receptors [17]. As the main ingredient of Fuzi, aconitine improves atrioventricular conduction, restores sinus rhythm, pressurizes, and dilates vessels with significant efficacy [18]. Moreover, aconitine can promote atrioventricular conduction by strengthening the excitability of the SAN and atrioventricular node [19]. In clinical practice, SFI is applied routinely in treating cardiac disorders, including cardiac arrhythmia, coronary heart disease, and heart failure [20–23]. In our previous study, we found that SFI has obvious clinical improvement effects on bradyarrhythmia [24], but the mechanism by which SFI exerts protective effects against SSS has not been fully elucidated. Therefore, our study established a rabbit SSS model to demonstrate the therapeutic effect of SFI treated-BMSCs and thoroughly examine the specific mechanisms.

## 2. Materials and Methods

### 2.1. Drugs and Reagents

SFI was gained from Ya'an Sanjiu Pharmaceutical Co., Ltd. (Ya'an, China) (specification: 10 mL/vial, approval number: National Medicine Standard Z51020664). Formaldehyde solution was gained from China National Medicines Co., Ltd. Penicillin G sodium was acquired from North China Pharmaceutical Co., Ltd. The HCN4 inhibitor, ZD7288 was purchased from MedChemExpress (HY-101346, USA), was dissolved to 0.1 mM in saline.

### 2.2. Rabbit BMSC Cultures

All animal procedures were carried out with the approval of the institutional animal care and use committee at the first affiliated hospital of Guangzhou University of Chinese Medicine (Guangzhou, China), and this study was performed in strict compliance with the *Guide for the Care and Use of Laboratory Animals of the National Institutes of Health* (NIH Publication No. 85-23, revised 1996). New Zealand rabbit bone marrow was collected in a 15 mL centrifuge tube under aseptic conditions, followed by suspension in 5 mL of phosphate buffer solution (PBS) and sieved through a 100-mesh filter. The filtrate was separated by density centrifugation at ambient temperature for 20 minutes at a rate of 1500 rpm through 5 mL of Ficoll-Paque solution (GE Healthcare, Madison, WI, USA). The interphase of the mononuclear fraction was recovered and subject to rinse two times in sterile PBS. After centrifugation, the pellet was subjected to resuspension, followed by planking into a 25 cm^2^ culture flask with 1 × 10^5^ cells. BMSCs were cultured in Dulbecco's Modified Eagle Medium/Nutrient Mixture F-12 (DMEM/F12) (Gibco, USA) that contained 15% fetal bovine serum (FBS), 100 U/mL penicillin, and 100 *μ*g/mL streptomycin in a humidified incubator with 5% of CO_2_ and at a temperature of 37°C. The culture medium was renewed with a fresh medium every three days, and the nonadherent cells were eliminated. BMSCs at passages 3 to 5 (P3-P5) were used in this study.

### 2.3. Flow Cytometry

Isolated BMSCs grown to 80-90% confluence were obtained by means of trypsinization and separated by centrifugation at a rate of 1000 rpm for 5 minutes, followed by rinse twice using PBS and transferring into EP tubes. Then, BMSCs were incubated with primary antibodies against HLA-DR, CD31, CD105, CD34, CD90, CD45, CD44, and CD29 for 30 minutes at 4°C. Finally, the cells were centrifuged and resuspended in PBS and conducted flow cytometer analysis (BD Biosciences, USA).

### 2.4. Transfection of BMSCs with HCN4

pLenti-GIII-CMV-GFP-2A-Puro-puc57-HCN4-RAb and pLenti-GIII-CMV-GFP-2A-Puro-Blank vector (lenti-GFP) were procured from Applied Biological Materials (ABM, Zhenjiang, China). BMSCs grown to 70-80% confluence were transfected with lentiviral HCN4 at a multiplicity of infection (MOI) of 100, and BMSCs treated with lenti-GFP were employed as the control. The medium was replaced with fresh complete medium 24 hours after transfection. After 24 hours, the transfected cells were visualized by a fluorescence microscope to identify the transfection rate. Ultimately, cells were incubated for an additional two days for the purpose of observing their morphology.

### 2.5. Cell Viability Assay

Cell Counting Kit-8 (CCK8, Dojindo, Japan) was employed for the purpose of examining the viability of BMSCs. First, BMSCs were seeded into 96-well plates. After different treatments (5, 10, 20, 40, or 80 *μ*L/mL SFI for 24 h), cells were exposed to CCK8 (10 *μ*L/well), followed by culturing at a temperature of 37°C for 4 hours. Finally, the absorbance of the cells was calculated at 450 nm using a Beckman AU480 microplate reader (Fullerton, CA, USA).

### 2.6. Transmission Electron Microscopy (TEM)

The morphology and ultrastructure of BMSCs were visualized using TEM as previously described [25]. Briefly, after treatment, BMSCs were washed with PBS three times, followed by fixing instantaneously in 2.5 percent glutaraldehyde in PBS for 2 hours at 4°C and a postfixation in 1 percent osmium tetroxide in 0.15 M cacodylate for 1 hour, rinsing with deionized water, dehydration in a graded series of ethanol (30, 50, 70, and 100 percent), embedding in 100 percent Spurr's resin, and polymerization for 48 hours at 60°C. Subsequently, ultra-thin segments (approximately 70 nm) of BMSC sections were cut using a diamond knife, mounted onto the copper grid, and visualized by TEM (H-7650, HITACHI, Japan) when magnified to the required extent.

### 2.7. Rabbit SSS Model and BMSC Transplantation

Healthy adult New Zealand rabbits (30 male and 30 female), weighing approximately 2 kg, were gained from Guangdong experimental animal center (Certificate No. SCXK [Guangzhou] 2018-0092). Animals were placed in the animal care facility and fed standard laboratory dietary foods twice a day. They received an unrestricted supply of tap water and were kept on a 12-hour dark/light rotation over the experimental duration. The animals were classified into six different groups depending on their average body weight, which was determined using a random number table, and were randomized to the following groups: sham-operation (Sham), SSS (model), SSS + BMSCs-GFP (GFP), SSS + BMSCs-HCN4 (HCN4), SSS + BMSCs-SFI-low (SFI-L), SSS + BMSCs-SFI-medium, (SFI-M) and SSS + BMSCs-SFI-high (SFI-H) groups.

The rabbits were immobilized on the examination table after being anesthetized intravenously with pentobarbital sodium (30 mg/kg, Sigma, USA), and the skin of their chests was aseptically prepped prior to surgery. The heart rate was continuously monitored by a surface electrocardiogram using the BL-420F data acquisition and analysis system. Subsequently, a thoracotomy was conducted through the right second-third intercostal region, during which a tiny portion of the right third rib was removed for the purpose of accurately locating the right auricle of the heart. The SAN, which is positioned at the intersection of the superior vena cava and the right atrial appendage, regulates the heart rhythm. Then, a cotton bud (with a diameter of 5 mm) dipped into 20% sterile formaldehyde solution was applied onto the SAN area (between the right auricle and the superior vena cava). Although 2 mg of atropine sulfate (Tianjin Jinyao Amino Acid, China) was administered intravenously, the heart rate remained 30-50 percent lower than it had been preoperatively, and it was not possible to restore it. Moreover, the animals displayed arrhythmia-like nodal escape, sinoatrial block, or sinus arrest, suggesting that the SSS model was successfully established. Hence, we kept the rabbits under observation for 2 hours to ensure that their heart rates remained stable. Moreover, the thoracic cavity was cleaned with sodium penicillin. Subsequently, different treatment BMSCs were prepared in the cell suspension. In the control group, BMSCs (1 × 10^7^ cells in 1 mL of PBS) were transplanted into the head, body, and tail of the SAN tissue (0.1 mL into each region) using a 1 mL needle. In the SFI group, BMSCs were treated with varying concentrations (10, 20, and 40 *μ*L/mL) of SFI, and the processed BMSCs were injected into the SAN area. Then, the thorax was closed, and the rabbits were taken back to the animal house. The rabbits in each group were subjected to euthanasia by CO_2_ asphyxiation one week following the procedure when the last heart rates were calculated.

### 2.8. Masson's Trichrome Staining

Excised SAN tissues were stained using Masson's trichrome staining to display the fibrotic area [26]. After removing the SAN area, sagittal sections were obtained, fixed in 10% formalin after dissection, followed by embedding in paraffin and slicing at a thickness of 5 *μ*m. Then, we performed deparaffinization of the paraffin sections and washing sequentially with distilled water, before staining with commercial reagents for Masson's staining (G1346, Solarbio, China), and then viewed and photographed under a light microscope (BX43, OLYMPUS, Japan). The fibrotic area was stained blue, and the myocardium area was stained red. The fibrosis area was calculated by Image-Pro software (Media Cybernetics Inc., Rockville, MD, USA).

### 2.9. Terminal Deoxynucleotidyl Transferase dUTP Nick-End Labeling (TUNEL) Assay

The apoptosis status of the SAN was determined utilizing the one-Step TUNEL Apoptosis Assay Kit (C1088, Beyotime Institute of Biotechnology, China). Sections of 5 *μ*m thickness were subjected to incubation for 30 minutes in 50 *μ*g/mL proteinase K solution. After being washed in PBS thrice (5 minutes for each wash), incubation of the sections was carried out in the TUNEL reagent for 2 h at a temperature of 37°C, followed by washing twice in PBS. 4,6-Diamino-2-phenyl indole (DAPI) was utilized for nuclei staining. After that, the sections were observed on antifluorescence quenching mounting tablets under a fluorescence microscope (CKX53, OLYMPUS, Japan). TUNEL staining of apoptotic cells is red, whereas DAPI staining of nuclei is blue. The percentage of apoptotic rate was calculated as red divided by the number of blue cells per field.

### 2.10. Immunohistochemistry

Gap junction proteins, namely, connexin 43 (Cx43) and 45 (Cx45) were checked by immunohistochemistry. When the deparaffinization was completed, the sections were microwave-heated in EDTA buffer (pH 9.0) for antigen extraction. Once the slides had been blocked with 10 percent goat serum, they were subjected to incubation using primary antibodies (Cx45, 1 : 50, Sigma, USA; Cx43, 1 : 300, bs-0651R, Bioss, China) at a temperature of 4°C throughout the night. After washing using PBS twice, the segments were subjected to incubation for 1 hour with the HRP-labeled goat anti-rabbit IgG (Nakasugi Jinqiao, Beijing, China). Then, the segments were subjected to incubation with DAB reagent (3,3′-diaminobenzidine) and visualized under an inverted microscope (BX43, OLYMPUS, Japan) for analysis.

### 2.11. Immunofluorescence Microscopy

The SAN tissues samples were inserted into optimal cutting temperature (OCT) compound (Sakura Fine technical Co., Ltd., Tokyo, Japan) and then frozen at -20°C. Frozen sections (8 *μ*m) were rinsed two times with PBS, and GFP was visualized in high-power fields under an immunofluorescence microscope (Nikon, Co., Tokyo, Japan).

### 2.12. RNA Isolation and Real-time Time qPCR

By following the instructions stipulated by the manufacturer, total RNAs were collected from SAN tissues or BMSCs using TRIzol Reagent (CW0580, CWBIO, China). Then, with the aid of the SuperScript First-Strand Synthesis Kit (CW2569M, Cwbio), the cDNA was obtained. The primers presented in Table 1 were acquired from General Biosystems Co., Ltd. (Anhui) in this experiment. Real-time PCR was conducted using 20 *μ*L of the reaction solution with 8.2 *μ*L RNase-free ddH_2_O, 10 *μ*L of 2× SYBR Green PCR Master Mix (Vazyme, Nanjing, China), and 0.8 *μ*L of primers mix, and 1 *μ*L cDNA. PCR was conducted in 96-well plates using the CFX-Connect Real-Time PCR system (Bio-Rad, Hercules, CA, USA) on the basis of the cycling conditions as follows: initial denaturation for 5 minutes at 95°C, followed by 40 cycles of 10 s denaturation at 95°C and annealing for 30 s at 58°C. The normalization of the expression levels of target genes to those of GAPDH was conducted, and the 2-^*ΔΔ*Ct^ method was employed to derive the fold change.

### 2.13. Western Blot

The western blot analysis was carried out in the same manner as discussed previously. After different treatments, total protein was isolated from BMSCs or SAN. For BMSCs, cells were subjected to rinsing using PBS and lysis on ice for 15 minutes with RIPA buffer (Beyotime Institute of Biotechnology) comprising a protease inhibitor cocktail (1 : 100). For SAN tissue, approximately 50 mg of frozen SAN was homogenized using a Quick Grinding Machine (60HZ, 60s, Tiss12, Shanghai Jingxin Industry) in 1 mL RIPA buffer with a protease inhibitor cocktail. After centrifugation at 12,000 × g for 10 minutes at a temperature of 4°C, the protein concentration was derived utilizing a protein assay kit (CW Biotech, China) for 30 min at 37°C. Proteins incubated with lysates (20 *μ*g) were isolated using 10 percent SDS-PAGE gels and then loaded onto polyvinylidene difluoride (PVDF) membranes. Subsequently, the TBST comprising 5 percent (w/v) skimmed milk was utilized for the purpose of blocking the membranes for 1 hour at ambient temperature. Moreover, the membranes were subjected to incubation at 4°C throughout the night with primary antibodies against HCN4 GAPDH (1: 2000, TA-08, ZSGB-Bio, China) and (1 : 1000, bs-1691r, Bioss, China). Eventually, the membranes were subjected to incubation for 1 hour at ambient temperature with the aid of the appropriate secondary antibodies (ZB-2305, ZSGB-BIO, China), after exposure to a film detection of the signals which was performed using the ECL Chemiluminescent Substrate Reagent Kit (Thermo, USA).

### 2.14. Enzyme-Linked Immunosorbent Assay (ELISA)

The levels of cAMP in the cell supernatants after different treatments were determined in accordance with the instructions stipulated by the manufacturer employing commercially available ELISA kits (020602, Mmbio, China). Cell supernatants were collected in tubes, centrifuged for 15 minutes at a rate of 2000 rpm, 4°C to eliminate the cell debris, followed by transferring into new tubes. Samples or cytokine standards were added to a precoated 96-well plate (50 *μ*L/well), and the dilution buffer was set as the blank. 100 *μ*L of the enzyme conjugate was added to normal and sample wells, but not the blank well, and the wells were sealed using an adhesive strip followed by incubation at a temperature of 37°C for 60 minutes. The plates were rinsed with 1× washing buffer (five times, 30 s each time), and substrate A solution (50 *μ*L/well) and substrate B solution (50 *μ*L/well) were introduced into the wells, followed by incubation at 37°C for 15 minutes in darkness. 50 *μ*L of the stop solution was added into each well to immediately halt the enzyme-substrate reaction. Then, the absorbance (OD) was tested at 450 nm, and the results were calculated by subtracting the blanks from the standards.

### 2.15. Whole-Cell Patch-Clamp Technique

The currents of the HCN4 channel were measured utilizing the whole-cell patch-clamp approach in a manner that has been previously described [27, 28]. BMSCs were detached using trypsin and subcultured onto glass coverslips for a minimum of 30 minutes prior to patch clamping. The whole-cell patch configuration was used in combination with the voltage clamp approach, which was carried out at ambient temperature (23-26°C). Micropipettes were pulled from borosilicate glass capillaries having an internal diameter of 0.89 mm, an external diameter of 1.5 mm, and a length of 10 mm. With regards to the whole-cell patch-clamp experiments, the diameter of the electrode tip is generally approximately 2 *μ*m, and the impedance is approximately 3-5 M*Ω*. The external solution for HCN4 currents was composed of the following: 10 mM glucose, 1.2 mM CaCl_2_, 1 mM MgCl_2_, 5 mM KCl, 135 mM NaCl, and 10 mM HEPES, pH 7.4. For the purpose of obtaining the Cs + external solution, 5 mM of cesium chloride (CsCl) was introduced to the external solution. The following was the components in the pipette solution: 10 mM EGTA, 10 mM HEPES, 3 mM MgCl_2_, 135 mM KCl, and 4 mM Na_2_.ATP, pH 7.35. The currents were recorded at ambient temperature utilizing a patch-clamp amplifier (Model 2400, A-M Systems, Carlsborg, WA, USA). Subsequently, signals at 2 kHz were low-pass filtered, and signals at 5 kHz were digitalized utilizing Axon 700B and pCLAMP software (version: 10.7) (both provided by Axon Instruments/Molecular Devices in Union City, California, United States). For the purpose of investigating the kinetic behaviors of channel activation, several hyperpolarizing step commands (from -70 mV to -150 mV with a 10 mV increment, every step lasting 3 s) were administered with the holding voltage set at -70 mV. To create the activation curve, the Cs + sensitive current recorded at each voltage step (*I*) was subjected to standardization to the peak current (*I*_max_ at -150 mV), followed by the plotting of the *I*/*I*_max_ was and fitting with the Boltzmann equation: *I*/*I*_max_ = 1/[1 − exp ((V1/2 − V)/*k*)]. We used a selective HCN4 channel blocker (50 *μ*M, ZD7288), to evaluate the effect on the HCN4 currents.

### 2.16. Statistical Analysis

The analysis results were presented as the mean ± standard error of the mean (SEM). Student's *t*-test was used to evaluate whether there were statistically significant differences between the two groups. Utilizing analysis of variance (ANOVA) with Tukey's post hoc test, we performed a comparison of the differences between groups. The threshold value for the significant level was fixed at *P* < 0.05. GraphPad Prism (version: 8.3) (GraphPad Prism Software Inc., San Diego, CA, USA) was used to carry out all of the statistical investigations.

## 3. Results

### 3.1. Characterization of BMSCs Transfected with LV-HCN4

**Figure 1:**
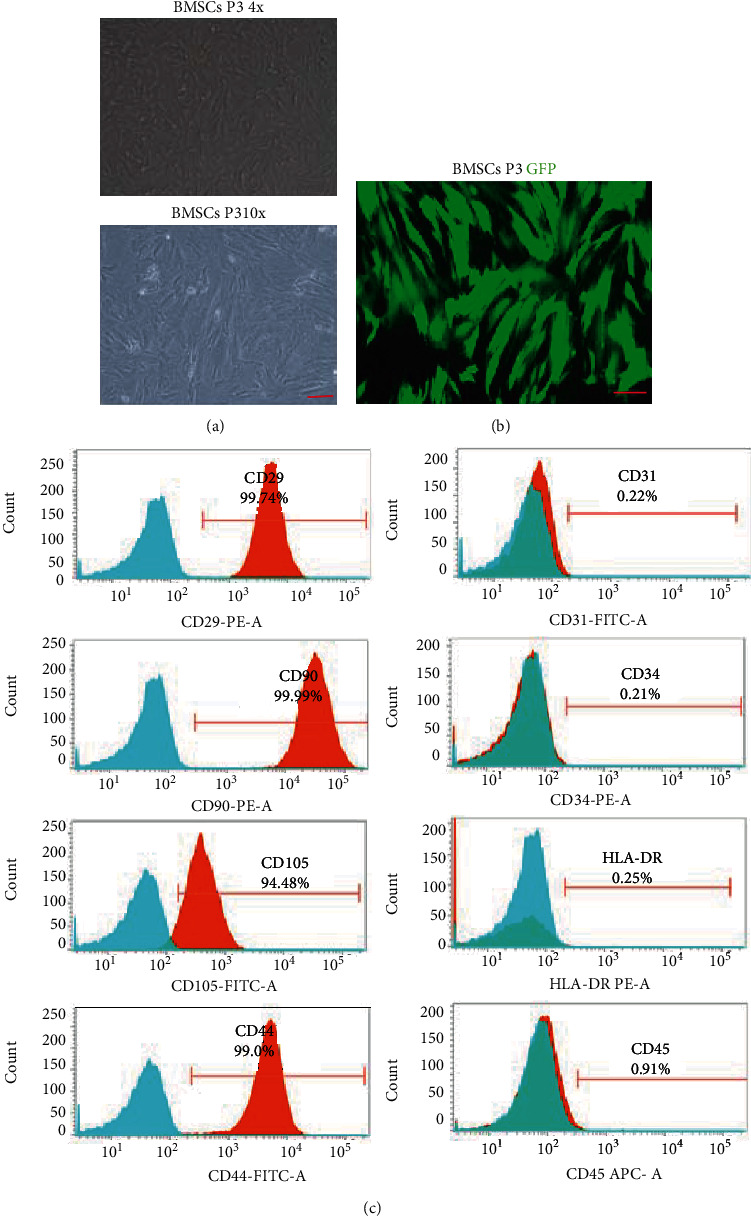
Characteristics and surface markers of bone marrow mesenchymal stem cells (BMSCs). (a) Morphological features of BMSCs exhibiting fibroblast-like morphology under microscopic observations. Scale bar = 100 *μ*m. (b) The expression of the fluorescent protein was observed under the fluorescence microscope. GFP: green fluorescent protein. Scale bar = 100 *μ*m. (c) Flow cytometry was used to detect the expression of surface markers of BMSCs. Positive markers CD90, CD105, CD29, and CD44 as well as negative markers CD45, HLA-DR, CD34, and CD31 were identified on the surface of BMSCs.

Primary cultured BMSCs displayed an adherent, fibroblast-like morphology under microscopic observations (Figure 1(a)). The result of flow cytometric analysis showed that the percentages of CD29-positive cells, CD44-positive cells, CD105-positive cells, and CD90-positive cells were 99.74%, 99.0%, 94.48%, and 99.99%, respectively (Figure 1(b)). These data indicated that purified BMSCs were successfully obtained. Transfection of BMSCs with lenti-HCN4 was performed once the cell confluence of 70-80% was attained and lenti-GFP, and the number of fluorescent cells (GFP) increased significantly at 72 h (Figure 1(c)), indicating that BMSCs were successfully transfected with HCN4.

### 3.2. SFI Promotes the BMSC Differentiation into Pacemaker-Like Cells

**Figure 2:**
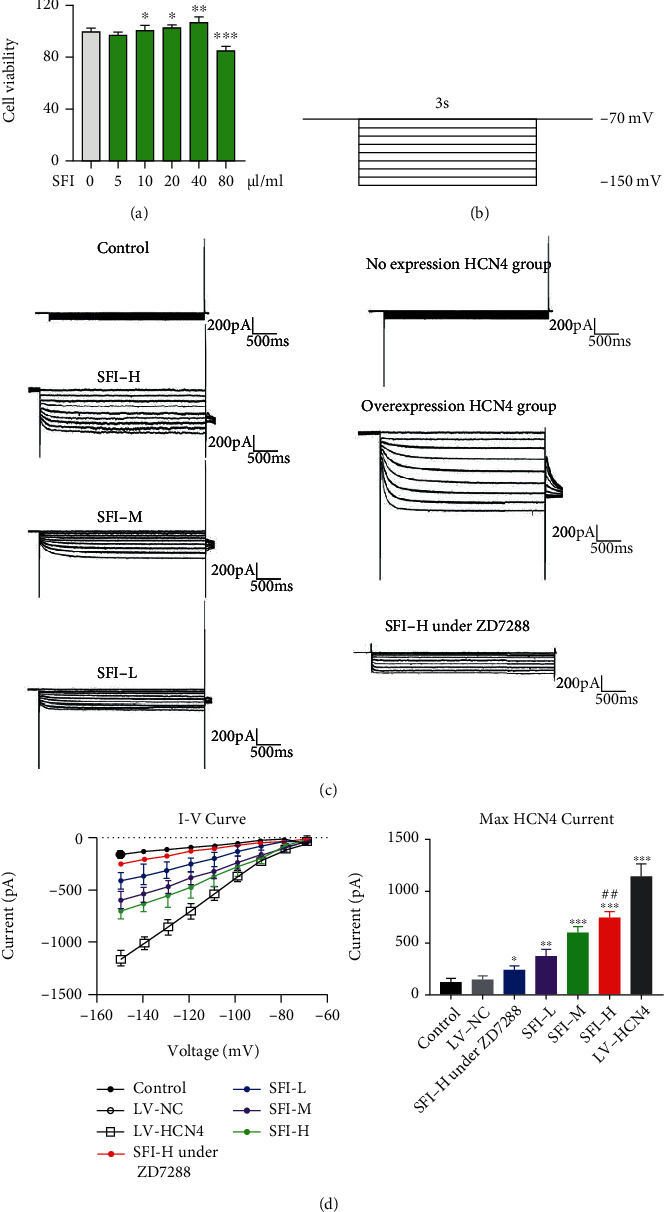
Shenfu injection (SFI) promotes the differentiation of BMSCs into pacemaker-like cells. (a) Cell Counting Kit-8 was utilized to detect the effect of SFI on the viability of BMSCs. ^∗^*P* < 0.05, ^∗∗^*P* < 0.01, ^∗∗∗^*P* < 0.001 vs. 0 *μ*L/mL. (b) BMSCs were clamped at -70 mV and stimulated with hyperpolarizing voltages ranging from -70 to -150 mV for 3 s in 10 mV steps to record cellular *I*_*f*_ currents. (c) Whole-cell patch-clamp electrophysiology was conducted and used for the purpose of recording the *I*_*f*_ current on BMSCs with different treatments. (d) SFI treatment could induce the expression of HCN4 channel in BMSCs. ^∗^*P* < 0.05 vs. LV-NC, ^#^*P* < 0.05 vs. SFI-H under ZD7288. Data represented mean ± SEM, *n* = 6. SFI-L: Shenfu injection-low dose (10 *μ*L/mL); SFI-M: Shenfu injection-medium dose (20 *μ*L/mL); SFI-H: Shenfu injection-high dose (40 *μ*L/mL).

For the purpose of observing the differentiation of BMSCs into pacemaker-like cells, we first used CCK8 to delve into the impact of SFI on the BMSCs viability and found that SFI at 10, 20, and 40 *μ*L/mL can promote the activity of BMSCs (Figure 2(a)). Then, whole-cell patch-clamp electrophysiology was performed to record the *I*_*f*_ current on BMSCs (Figure 2(b)). BMSCs in the HCN4 overexpression group were found to express a larger hyperpolarized inward pacing current than normal BMSCs under the same gradient voltage stimulation, and BMSCs in the SFI treatment group were also found to activate the inward pacing current (Figure 2(c)). Moreover, the higher the concentration, the stronger the current and highly sensitive to HCN4 channel-specific blocker ZD7288 (Figure 2(d)). These data indicate that SFI promotes the opening of the HCN4 current channel and activates the *I*_*f*_ current, thereby inducing the BMSC differentiation into pacemaker-like cells.

### 3.3. Expression of HCN4 in SFI-Treated BMSCs

**Figure 3:**
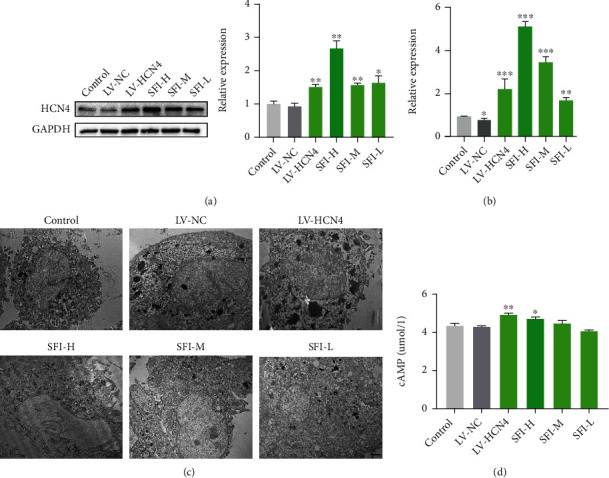
Expression of HCN4 in BMSCs treated with Shenfu injection (SFI). (a) Western blot was conducted to examine the HCN4 expression in SFI-treated BMSCs at protein levels. (b) Real-time PCR was used to detect HCN4 mRNA expression. Data are expressed as the mean ± SEM, *n* = 3 − 6, ^∗^*P* < 0.05, ^∗∗^*P* < 0.01, ^∗∗∗^*P* < 0.001 vs. LV-NC. (c) Representative transmission electron microscopy (TEM) images of the ultrastructure derived from BMSCs. Scale bar = 2 *μ*m. (d) ELISA was performed to detect the expression of cAMP. Data are expressed as the mean ± SEM, *n* = 6, ^∗^*P* < 0.05, ^∗∗^*P* < 0.01 vs. LV-NC. SFI-L: Shenfu injection-low dose (10 *μ*L/mL); SFI-M: Shenfu injection-medium dose (20 *μ*L/mL); SFI-H: Shenfu injection-high dose (40 *μ*L/mL).

To examine the mechanisms that underlie the differentiation of BMSCs into pacemaker-like cells, qPCR and western blotting were conducted to test the expression profile of HCN4, which significantly increased in BMSCs treated with SFI after 72 h in culture (Figures 3(a) and 3(b)). TEM analysis confirmed SFI can improve the cell ultrastructure of BMSCs (Figure 3(c). In the control group and LV-NC group, the nucleoli and nuclear membranes of BMSCs were clear. In contrast with the control group, the LV-HCN4 group had larger nuclei, a more complete membrane, and a remarkable elevation in the levels of the stromal vesicles, mitochondria, and rough endoplasmic reticulum. In the SFI-treated group, stromal vesicles, mitochondria, and rough endoplasmic reticulum were increased, and cells were expanded. The degree of cell improvement in the SFI-treated group was elevated in direct proportion to the dosage of SFI administered. In contrast with the control group, the SFI group had a substantially elevated cAMP content, and the higher the concentration of SFI, the more obvious the increase in cAMP (Figure 3(d)). These results showed the increased expression of HCN4 in BMSCs treated with SFI.

### 3.4. Effect of SFI-Treated BMSCs on SAN Tissue

**Figure 4:**
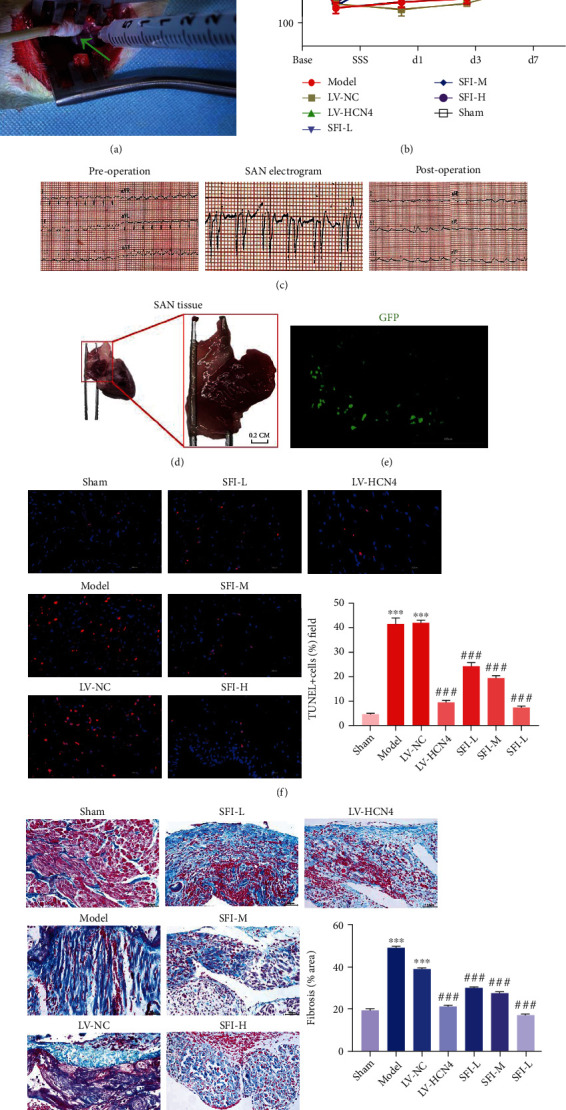
Impacts of SFI-treated BMSCs on the sinoatrial node (SAN) tissue. (a) After establishing the rabbit model of sick sinus syndrome (SSS) with SAN dysfunction, green fluorescent protein- (GFP-) expressing BMSCs (1 × 10^7^ cells in 1 mL of PBS) were transplanted into the head, body, and tail of the SAN tissue. Photograph of the implantation procedure for transduced BMSCs. (b) The rabbits' mean heart rate prior to and after SFI-treated BMSC transplantation. Data are expressed as the mean ± standard error of the mean (SEM). bpm: beats per minute. ^∗^*P* < 0.05. (c) Typical electrocardiogram (ECG) image of the sinus rhythm before and after the transplantation of SFI-treated BMSCs, and the middle ECG represents the electrogram of the SAN. A relatively small QRS and deep reversed P wave were observed in the ECG. (d) Tissue of the SAN. (e) Microscopic images of GFP-expressing BMSCs transplanted into the SAN tissue of rabbits with SSS. (f) TUNEL assay was performed to detect apoptosis of SAN cells. Scale bar = 100 *μ*m. Data are expressed as the mean ± SEM. ^∗∗∗^*P* < 0.001 vs. sham; ^#^*P* < 0.05 vs. LV-NC. (g) Masson's trichome staining was conducted to detect local fibrosis in the SAN tissue. The area of blue represented the fibrotic tissue. Scale bar = 50 *μ*m. Data are articulated as the mean ± SEM. ^∗∗∗^*P* < 0.001 vs. sham; ^#^*P* < 0.05 vs. LV-NC. SFI-L: Shenfu injection-low dose (10 *μ*L/mL); SFI-M: Shenfu injection-medium dose (20 *μ*L/mL); SFI-H: Shenfu injection-high dose (40 *μ*L/mL).

We used the formaldehyde wet compressing method to establish the SSS rabbit model (Figure 4(a)). We recorded the heart rate of the rabbits on days 1, 3, and 7 after the surgery. Assessment of real-time ECG monitoring revealed that the mean heart rate was significantly reduced after the surgery. Interestingly, we found that the mean heart rate of the SSS rabbits in the SFI treatment group was significantly elevated in contrast with that of the SSS rabbits in the control group (Figures 4(b) and 4(c)). These results indicated support that SAN dysfunction was successfully induced in the SSS rabbit model via formaldehyde wet compressing. The SAN is positioned at the junction of the superior vena cava and the right atrial appendage (Figure 4(d)). Then, we injected GFP-expressing BMSCs into the SAN tissue successfully (Figure 4(e)). TUNEL images illustrated that the apoptosis rate of SAN in the sham group was extremely low, while that in the model group was high (*P* < 0.001). In contrast, the rate of apoptosis in the SFI treatment group was lower as opposed to that in the model group (Figure 4(f)), and the higher the concentration of SFI, the lower the rate of apoptosis. Results of Masson's trichome staining showed that the injection of SFI-treated BMSCs into the SAN substantially attenuated the fibrosis of the SAN in rabbits in contrast with the model group (Figure 4(g)).

### 3.5. Evaluation of the HCN4 Expression and Gap Junction Protein Expression in the SAN of BMSC-Transplanted Rabbits with SSS

**Figure 5:**
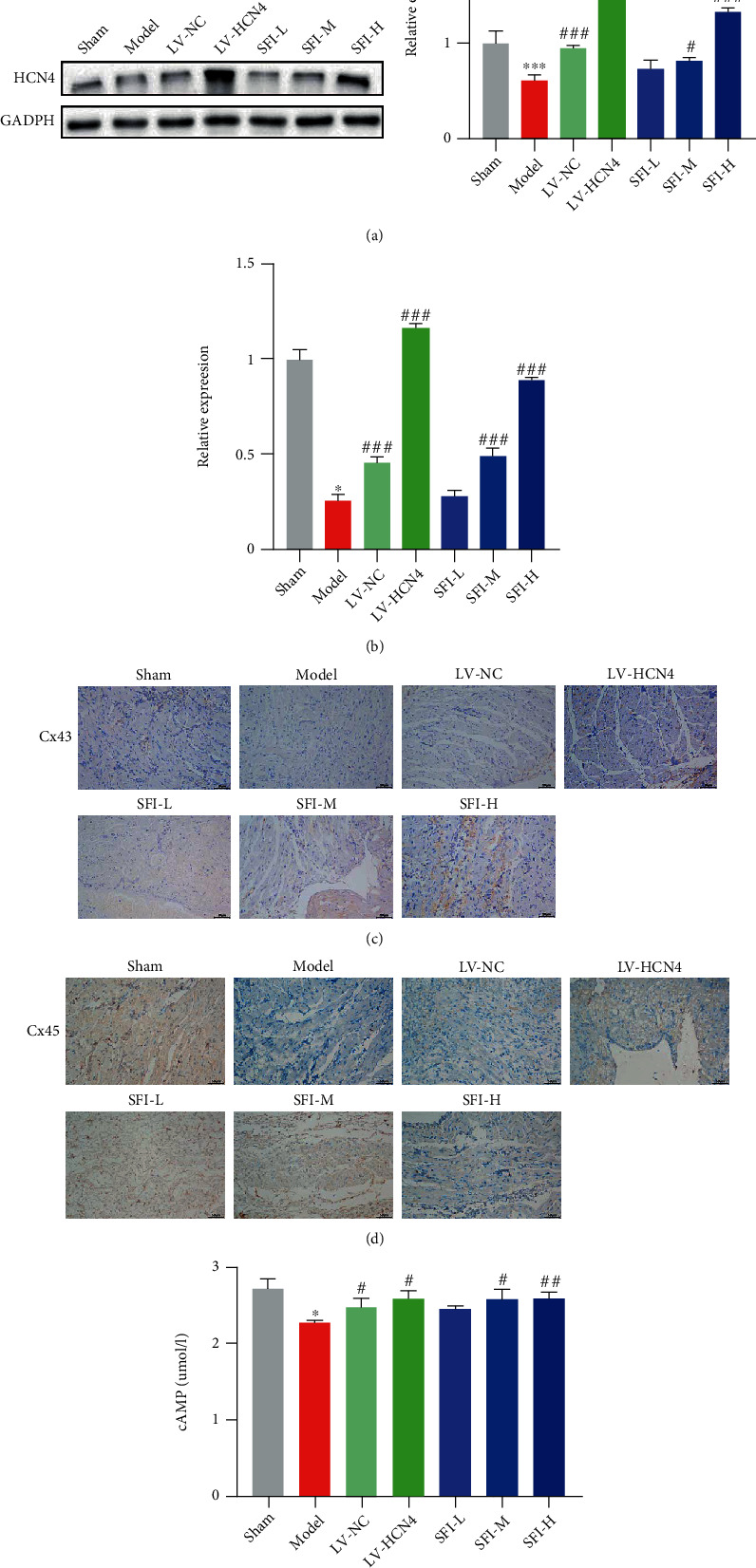
Evaluation of the HCN4 expression and gap junction protein expression in the SAN of BMSC-transplanted rabbits with SSS. (a, b) Detection of HCN4 in the SAN of BMSC-transplanted rabbits at protein and mRNA levels by real-time PCR and western blot, respectively. Data are expressed as the mean ± SEM. ^∗^*P* < 0.05, ^∗∗^*P* < 0.01 vs. sham; ^#^*P* < 0.05, ^###^*P* < 0.001 vs. LV-NC. (c, d) Immunochemistry was conducted to determine the Cx43 and Cx45 protein expression in the SAN tissue of the SSS rabbit model. Scale bar = 50 *μ*m. (e) ELISA was performed to detect the cAMP content in the SAN tissue of the SSS rabbit model. ^∗^*P* < 0.05 vs. sham; ^#^*P* < 0.05, ^##^*P* < 0.01 vs. LV-NC.

To investigate the mechanism of the protective effect of SFI-BMSCs against SSS in rabbits, we removed the SAN tissue of the rabbits and subjected them to western blot and qPCR for detection of HCN4. Based on the findings, the SFI-BMSCs group had remarkably elevated HCN4 expression in the SAN tissue in contrast with the model group (Figures 5(a) and 5(b)). The impact of SFI on the Cx43 and Cx45 protein expression in the SAN tissue of the SSS rabbit model was determined by immunohistochemistry. The findings illustrated that SFI could promote the Cx43 and Cx45 protein expression in the damaged SAN tissue (Figures 5(c) and 5(d)). Furthermore, compared with that in the model group, the SFI group exhibited a remarkably elevated cAMP content (Figure 5(e)). Taken together, these data demonstrate that the SFI-induced pacemaker activity was released from the SAN.

## 4. Discussion

**Figure 6:**
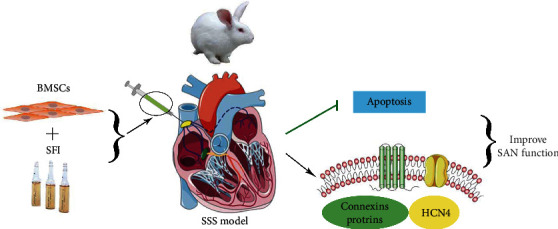
Transplantation of Shenfu injection-treated BMSCs into the sinoatrial node of rabbits with sick sinus syndrome.

In the present research, we investigated the function of SFI in the differentiation of BMSCs into pacemaker-like cells. The findings illustrated that the transplantation of SFI-treated BMSCs improved the pacing function of the SAN in SSS rabbits (Figure 6).

Patients suffering from bradyarrhythmia might gain benefit from the use of biological pacemakers that could be manufactured through gene or cell therapy to reestablish cardiac rhythm as well as conduction functionality [29]. As a stem cell-based therapy, BMSCs are widely used in basic and clinical research. MSCs were employed in combination with a self-inactivating lentiviral vector so as to transfer the human sodium/potassium hyperpolarization-activated cyclic nucleotide-gated ion channel 2 (HCN2) into rabbit bone marrow stem cells (BMSCs), according to a recent research report [30]. The canine heart was implanted with human mesenchymal stem cells (hMSCs) that had been subjected to transfection using the mouse HCN2 (*mHCN2*) gene for the purpose of establishing a biological rhythm [31]. In addition, some other types of stem cells were used to convert mature myocytes into SAN-like cells. The integration of TBX5 (SHT5), HCN2, and SHOX2 transcription factors and channel proteins has been suggested as a promising stem cell therapy for SSS and CPCs can be reprogrammed to serve as pacemaker-like cells using this approach [32]. Several research reports have demonstrated the forward programming of pluripotent stem cells (PSCs) with Tbx3 [33] or SHOX2 [34] and in vivo transformation of commonly existing contractible cardiomyocytes to pacemaker-like cells via the elevated Tbx18 expression, the main transcriptional factor involved in the progression of SAN [35]. Feng et al. developed an effective transgene-independent differentiation protocol for synthesizing sinoatrial node-like cells (SANLC) from hiPSCs, in which the combined modulation of RA, FGF, and BMP signaling at the cardiac mesoderm stage results in the generation of SANLC with great effectiveness. Hence, it could be a promising technique for the development of biological pacemakers [3]. Jian et al. stimulated the adipose tissue-derived stem cell (ADSCs) differentiation into cardiac pacemaker cells by means of the coexpression of the transcriptional factors T-box18 and insulin gene enhancer-binding protein 1 (ISL-1) [36]. Furthermore, Zhu et al. indicated that lncRNA modulates the ESC differentiation into pacemaker-like cells via the modulation of HCN4 [37]. Therefore, BMSCs have paved the way for the identification of cell-based biological pacemakers.

Traditional Chinese medicine, which incorporates treatment modalities with multitarget and multipathway effects, can regulate the differentiation, proliferation, and migration of MSCs to treat various diseases, such as liver fibrosis [38], cerebral ischemia-reperfusion [39], and osteoporosis [40]. SFI, an extensively recognized and essential type of Chinese traditional medicine, is utilized in treating cardiovascular disorders, such as dilating coronary arterioles, stabilizing blood pressure, alleviating hypoxia-reoxygenation injury, and improving heart function [21, 41–43]. Wang et al. showed that SFI could suppress programmed cell death by regulating the expression of caspase-3 and Bcl-2 in the process of hypoxia/reoxygenation in neonatal rat cardiomyocytes in vitro [21]. Additionally, SFI can attenuate rat myocardial hypertrophy by upregulating the miR-19a-3p expression and modulating the MEF2 signaling pathway [44]. In the present research, we utilized SFI to stimulate differentiation of BMSCs into pacemaker cells and transplanted the differentiated BMSCs to treat SSS. The results confirmed the effectiveness and safety of pacemaker-like cells differentiated from BMSCs in improving the pacing function of the SAN. Our research provides a new strategy for the construction of biological pacemakers.

In previous studies, several procedures for constructing SSS animal models have been introduced, mainly involving physical damage to the SAN, such as right coronary artery ligation, radiofrequency ablation, and condensation. In our study, the formaldehyde wet compressing method was used to establish the rabbit model of SSS. Based on extensive literature review and experiments [45–48], we transplanted BMSCs (1 × 10^7^ cells) into the head, body, and tail regions of the SAN tissue (0.1 mL into each region). In our previous study, we found that the transfection of cardiomyocytes in neonatal mice with HCN4 adenovirus vector might significantly increase the heart rate, thus clarifying the effect of HCN4 on cardiac pacing. In the present research, we found that SFI could promote the HCN4 gene expression and the BMSCs differentiation into pacemaker-like cells. However, the mechanism by which SFI promotes the transformation of BMSCs into pacemaker-like cells needs to be further studied in large animals.

## Figures and Tables

**Table 1:** Primer sequences of HCN4.

Gene	Primer sequence
HCN4	F:GCTGTCAAAGTGGAGGGAGG R:GCGAGAATTTGTTGACCCCG
GAPDH	F:CCACTTTGTGAAGCTCATTTCCT R:TCGTCCTCCTCTGGTGCTCT

## Data Availability

All the data generated or analyzed during this study are included in this published article.
